# Morphological Aspects in Ultrasound Visualisation of the Suprascapular Notch Region: A Study Based on a New Four-Step Protocol

**DOI:** 10.3390/jcm7120491

**Published:** 2018-11-27

**Authors:** Hubert Jezierski, Michał Podgórski, Grzegorz Wysiadecki, Łukasz Olewnik, Raffaele De Caro, Veronica Macchi, Michał Polguj

**Affiliations:** 1 Department of Orthopedics and Traumatology, Ministry of the Interior Hospital, Północna 42, 91-245 Łódź, Poland; hjez@o2.pl; 2 Department of Diagnostic Imaging, Polish Mother’s Memorial Hospital Research Institute, 81/289 Rzgowska, 93-338 Łódź, Poland; michal.podgorski@umed.lodz.pl; 3 Department of Normal and Clinical Anatomy, Medical University of Lodz, Żeligowskiego 7/9, 90-752 Łódź, Poland; grzegorz.wysiadecki@umed.lodz.pl (G.W.); lukasz.olewnik@umed.lodz.pl (L.O.); 4 Institute of Human Anatomy, Department of Neurosciences, University of Padova, Via A. Gabelli 65, 35127 Padova, Italy; rdecaro@unipd.it (R.D.C.); veronica.macchi@unipd.it (V.M.); 5 Department of Angiology, Medical University of Lodz, Żeligowskiego 7/9, 90-752 Łódź, Poland

**Keywords:** ultrasound, BMI, suprascapular notch, suprascapular neuropathy, suprascapular nerve blockade

## Abstract

Background: Sonographic evaluation of the suprascapular notch (SSN) region is clinically important, because it is the most common location for performing suprascapular nerve block. The aim of the study was to describe the morphology of the SSN region based on ultrasound examination and in accordance with the patients’ body mass index (BMI). Material and Methods: The SSN region was sonographically examined in 120 healthy volunteers according to our new four-step protocol. The morphometry of the SSN and the neurovascular bundle was assessed, and patients’ BMI were calculated. The shape of the suprascapular notch was classified based on its superior transverse diameter (STD) and maximal depth (MD). Result: The type III scapular notch was the most prevalent (64%). The BMI was higher in type IV/V (27.38 ± 3.76) than in type I (24.77 ± 3.49). However, no significant differences were observed in the distribution of SSN notch types with regard to BMI (*p* = 0.0536). The suprascapular artery was visualised in all of the recognised SSNs, while the suprascapular vein and nerve were visualised only in 74.9% and 48.1% of the SSNs, respectively. The suprascapular nerve was significantly thicker on the right side (3.5 ± 1.1 mm) than on the left (1.3 ± 0.4 mm) (*p* = 0.001). In contrast, the suprascapular vein (1.5 ± 0.9 mm) was found to be a significantly wider on the left side than the right (1.2 ± 0.7 mm) (*p* = 0.001). Conclusion: Our original four-step sonographic protocol enabled characterising the morphology of the SSN region, despite the SSN notch types. The suprascapular artery is the best sonographic landmark for the suprascapular notch region. No significant differences were found between sides regarding the thickness of the soft tissue above the suprascapular nerve and vessels. Recognition of the SSN morphology is not affected by the BMI.

## 1. Introduction

**Figure 1:**
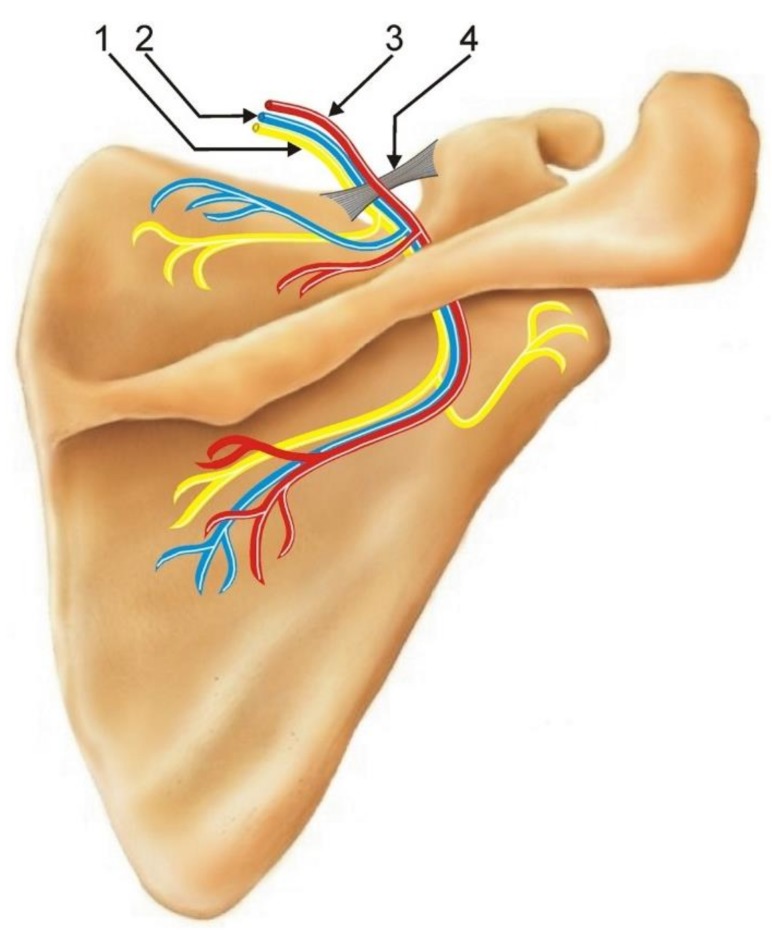
Schematic arrangements of structures passing around the suprascapular notch. 1: suprascapular nerve; 2: suprascapular vein; 3: suprascapular artery; 4: superior transverse scapular ligament (STSL).

The suprascapular notch (SSN) is a depression located on the superior border of the scapula, medially to the coracoid process. The notch is conversed into the opening by the superior transverse scapular ligament (STSL). Typically, the suprascapular nerve and vein run through the foramen formed below the STSL, and the suprascapular artery passes above the STSL (Figure 1). A great deal of variation exists in the SSN region regarding the morphology of the SSN itself [1,2], the morphology of the superior transverse scapular ligament [3,4] and anterior coracoscapular ligament [5,6], and the arrangement of the suprascapular triad (a nerve, a vein, and an artery) [7,8].

The morphology of the SSN region is important from a clinical point of view, because it is the most common site of suprascapular nerve compression and injury [9,10]. In addition, the development of suprascapular nerve entrapment follows a complex etiology that is also affected by the morphological variations in this region. For example, the presence of a narrow SSN and V-shaped notch may increase the likelihood of its occurrence [2,11]. Also, the band-shaped, bifid, or completely ossified STSL may be more likely to be associated with nerve entrapment [12,13,14]. Therefore, knowledge of the morphology of the SSN region, especially SSN shape and STSL variations, is particularly important in various procedures employing a sonographic evaluation of the SSN region.

Developments in ultrasound transducer technology have enabled the precise investigation of several anatomical structures compacted into small areas, these being nerves, vessels, or even small band-shaped fascicular structures [15,16,17]. Accurate sonographic visualisation of the nerve may also increase the success rate of the blocking procedures performed on it [18]. In addition, sonography is a useful tool for identifying some painful clinical conditions and preventing possible complications [19,20].

The aim of this study was to describe the morphological characteristics of the SSN region based on ultrasound examination and in the context of individual conditions (particularly body mass index, or BMI). It is a detailed study based on a new, original four-step (“step-by-step”) sonographic protocol for evaluation of the SSN region.

## 2. Material and Methods

The research project included 120 healthy volunteers (66 women and 54 men) from the Orthopedic Department of the Ministry of Internal Affairs Hospital in Lodz, Poland. The research project was approved by the Bioethics Commission of the Medical University of Lodz (Protocol number ID: RNN/586/14/KE). All of the procedures that took place in the study were in accordance with the ethical standards of the responsible committee on human experimentation (institutional and national) and with the Helsinki Declaration of 1975, as revised in 2008. All of the participants gave informed oral and written consent to take part. A sonographic examination was performed of the SSN notch region using a Toshiba memioXG (Toshiba, Japan) apparatus with 5–10 MHz linear transducer. The patient was placed in a sitting position in front of the researcher. The examination was performed according to our newly developed four-step protocol. In the first step, the probe was moved along the SSN in a parasagittal plane; in the second step, the probe was moved along the SSN in a paracoronal plane; in the third step, the probe was placed in the paracoronal plane and moved forward until reaching the upper edge of the scapula; finally, in the fourth step, Doppler ultrasound was used to recognise the suprascapular vessels [21]. The BMI of the patients was calculated based on measurements of their height and body mass in light clothing and without shoes. 

The exclusion criteria were as follows: fracture of the scapula, active neoplastic disease with metastases to the scapula, the presence of scars and wounds on the skin of the shoulder area, or the presence of deformations, injury, or operation of the shoulder region.

**Figure 2:**
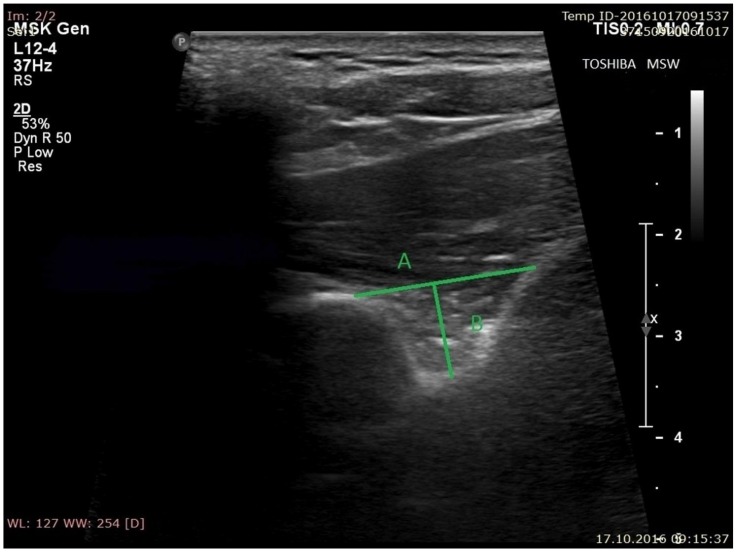
Measurements of suprascapular notch during ultrasonographic investigation. (**A**) the superior transverse diameter (STD); (**B**) the maximal depth (MD).

**Figure 3:**
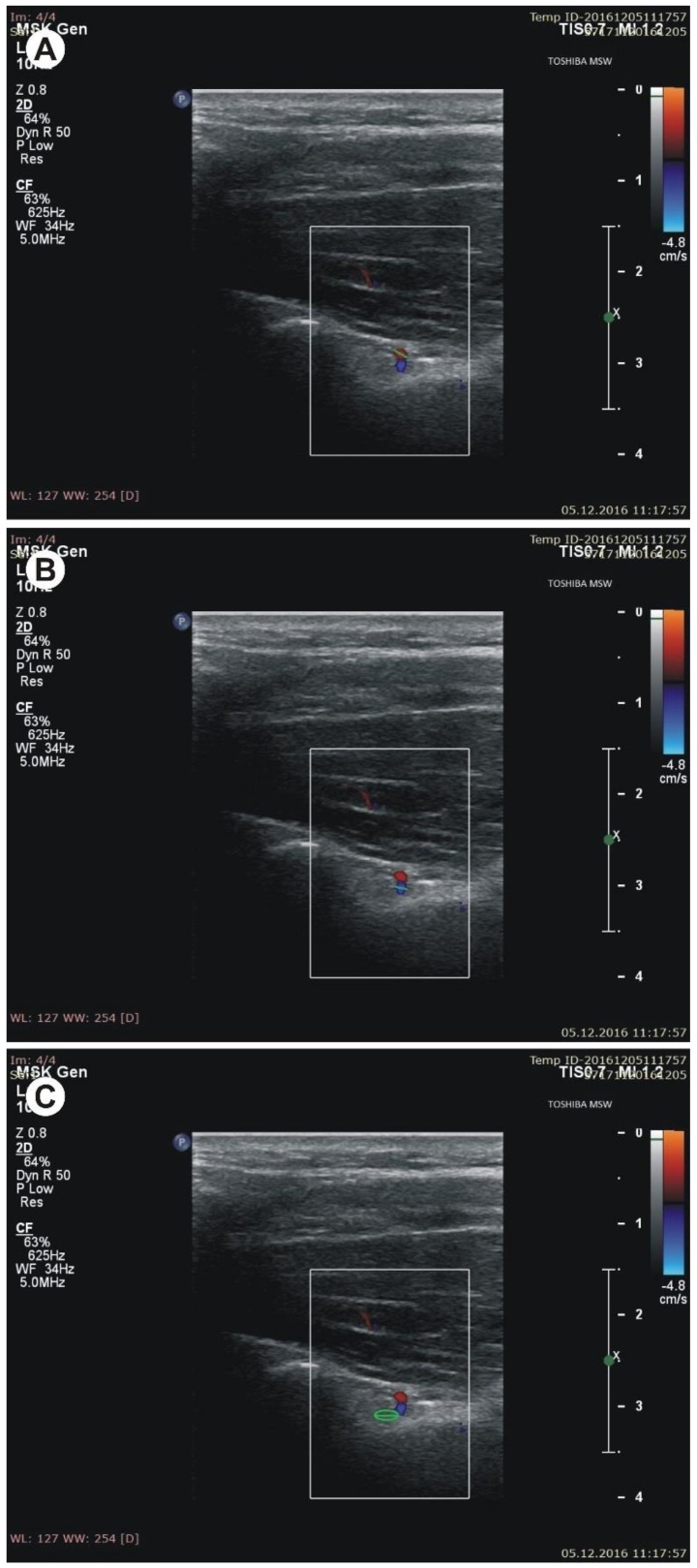
Measurements of structures at the suprascapular notch (SSN) notch region during sonographic examination. (**A**) diameter of the suprascapular artery; (**B**) diameter of the suprascapular vein; (**C**) diameter of the suprascapular nerve.

**Figure 4:**
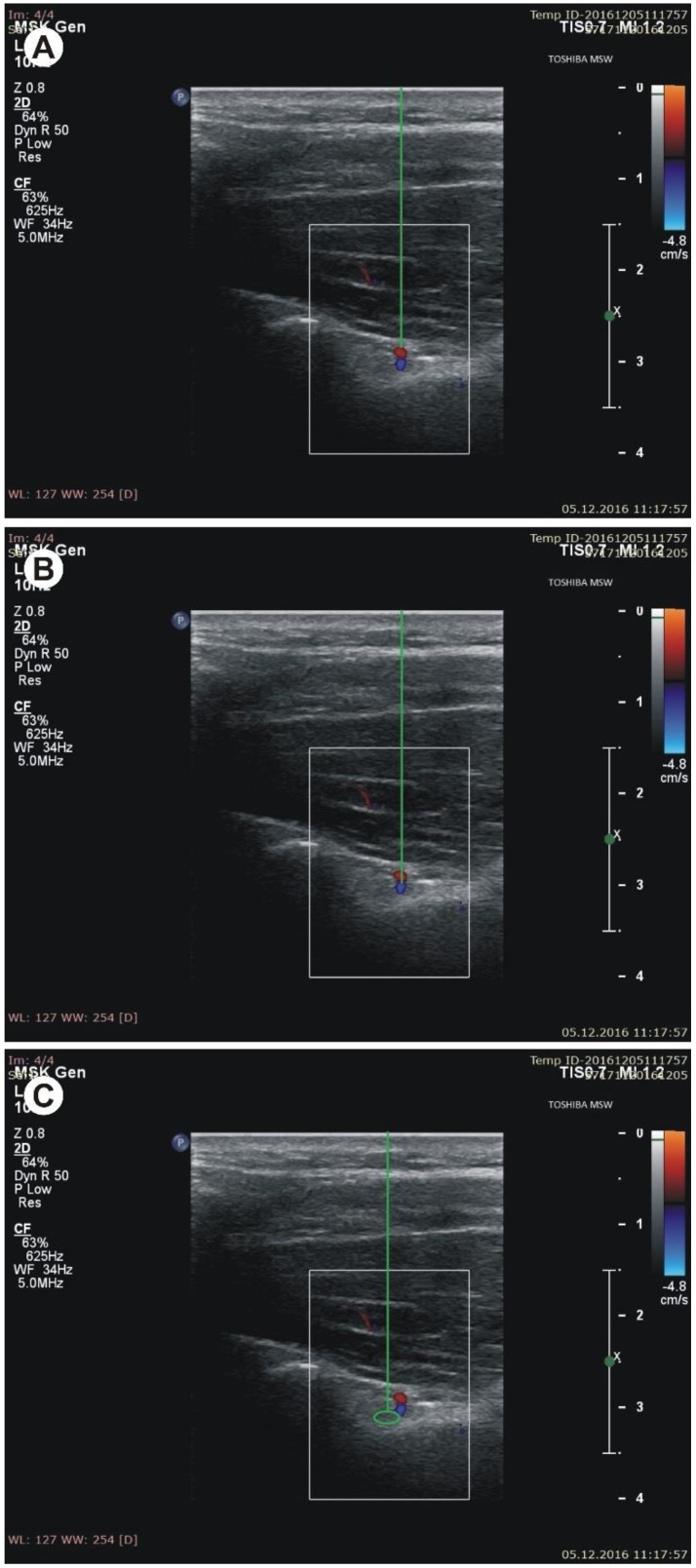
Measurements of the suprascapular notch region during ultrasonographic investigation. (**A**) the thickness of the soft tissue over the suprascapular artery; (**B**) the thickness of the soft tissue over the suprascapular vein; (**C**) the thickness of the soft tissue over the suprascapular nerve.

During sonographic investigation, the following measurements were collected:

(a) the superior transverse diameter (STD) of the suprascapular notch: the maximal distance in the horizontal plane between the corners of the suprascapular notch (Figure 2).
(b) the maximal depth (MD) of the suprascapular notch: the distance between the STD and the deepest point of the suprascapular notch measured in a plane perpendicular to the STD (Figure 2).
(c) the diameter of the suprascapular artery (Figure 3A)
(d) the diameter of the suprascapular vein (Figure 3B)
(e) the diameter of the suprascapular nerve (Figure 3C)
(f) the thickness of the soft tissue over the suprascapular artery: the minimal distance between the suprascapular artery and the skin (Figure 4A)
(g) the thickness of the soft tissue over the suprascapular vein: the minimal distance between the suprascapular vein and the skin (Figure 4B)
(h) the thickness of the soft tissue over the suprascapular nerve: the minimal distance between the suprascapular nerve and the skin (Figure 4C).

**Figure 5:**
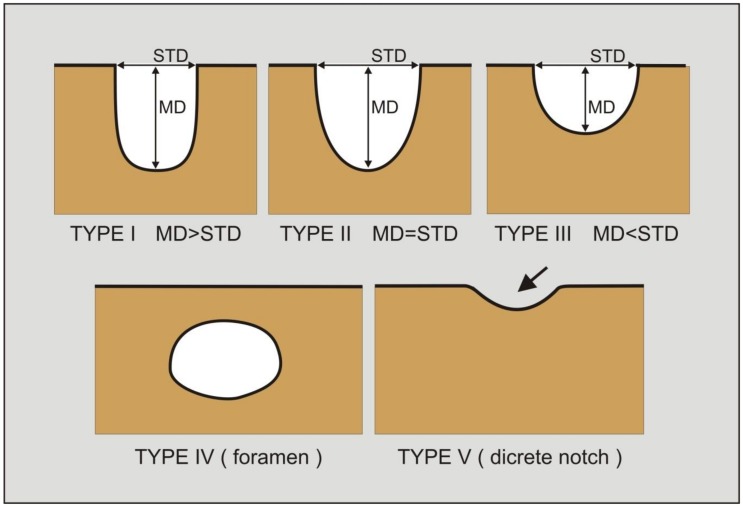
Classification of the suprascapular notch variations. MD: maximal depth of the suprascapular notch; STD: superior transverse diameter of the suprascapular notch. Type I: MD is longer than STD; Type II: MD and STD are equal; Type III: STD is longer than MD; Type IV: a suprascapular foramen as a bony foramen; Type V: a discrete notch (arrow).

The shape of the suprascapular notch was classified based on its superior transverse diameter (STD) and maximal depth (MD). The notches were classified as follows, according to Polguj et al. [17]: type I—the maximal depth is greater than the superior transverse diameter (MD > STD); type II—the maximal depth is equal to the superior transverse diameter (MD = STD); type III—the superior transverse diameter is greater than the maximal depth (STD > MD) (Figure 5). In addition, a fourth category entitled type IV/V was used for all of the notches in which only the bony margin was visualised, without any depression. In the original classification by Polguj et al. [17], type IV is defined as an arrangement where the superior transverse scapular ligament is ossified and a bony foramen is formed, while type V bears a discrete notch. As it was impossible to sonographically differentiate between types IV and V, notches in which only the bony margin was visualised without a depression were classified as “type IV/V” [21].

### Statistical Analysis

Statistical analysis was performed with Statistica 12.0 software (StatSoft, Cracow, Poland). For continuous variables, mean and standard deviation (SD) were provided. The Shapiro–Wilk test was used to test data for a normal distribution. For comparisons of nominal variables between groups, the Chi^2^ test was applied with contingency tables. As the obtained data was found to be not normally distributed, nonparametric tests were used for the comparison of continuous variables between two groups: the Mann–Whitney test for independent variables and the Wilcoxon sign rank test for dependent variables. To compare continuous variables between more than two groups, the Kruskal–Willis ANOVA with post hoc tests was applied. For assessment of correlations, the Spearman’s rank correlation coefficient was calculated. For multiple comparisons, Bonferroni’s correction was applied. A *p*-value of 0.05 or below was considered significant.

## 3. Results

The mean age of the 120 tested patients (54 men, 66 women) was 54.8 ± 15.5 years. The BMI was 26.3 ± 4.3 for women and 26.4 ± 3.2 for men (*p* = 0.9112). In 115 of the 120 patients, both SSN notches were fully visualised. In five patients, the scapular notch was unilaterally obscured by the clavicle, and could not be examined.

All of the SSN notches were classified into one of four types. As it was impossible to sonographically differentiate between types IV and V, these notches were merged into a shared classification of type IV/V. The distribution of the suprascapular notch types across the whole group was as follows: type I (11.1%), type II (6.0%), type III (64%), and type IV/V (18.7%). The subjects with type IV/V tended to have a higher BMI (27.38 ± 3.76) than those in type I (24.77 ± 3.49). However, no significant differences were observed in the distribution of SSN notch types with regard to BMI (*p* = 0.0536) (Table 1). 

**Figure 6:**
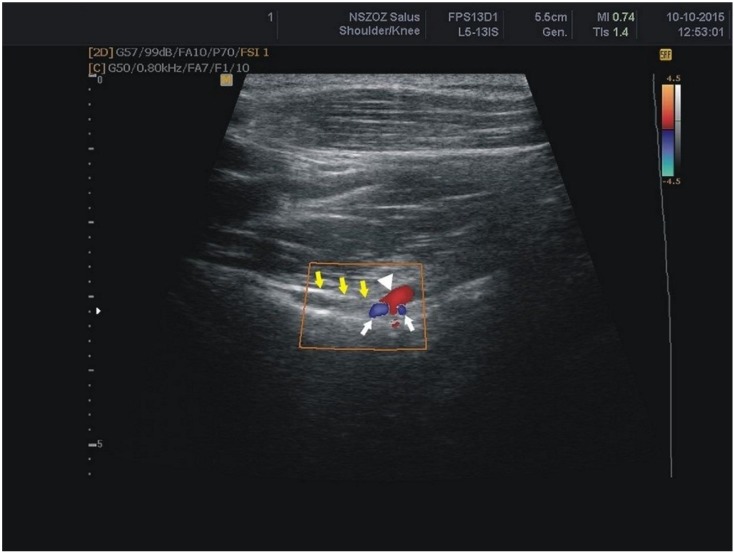
Sonogram of the suprascapular notch region (color Doppler) white arrows: suprascapular veins; arrowhead: suprascapular artery; yellow arrows: superior transverse scapular ligament.

The suprascapular artery was recognised in all of the visualised notches (Figure 6). The suprascapular vein was visible more often than the suprascapular nerve (74.9% versus 48.1%) (Figure 6) (Table 2). There was a significant correlation between the BMI and thickness of the soft tissue over the suprascapular artery and vein (Table 3).

The thickness of the soft tissue over the suprascapular triad (neurovascular bundles) was as follows: 34.2 ± 5.7 mm for the suprascapular artery, 35.8 ± 6.0 mm for the suprascapular vein, and 36.0 ± 5.7 mm for the suprascapular nerve (Table 3). No significant differences in these parameters were found between body sides (Table 4). The suprascapular nerve was significantly thicker on the right side of the body (3.5 ± 1.1 mm) than on the left (1.3 ± 0.4 mm) (*p* = 0.001) (Table 4). In contrast, the suprascapular vein (1.5 ± 0.9 mm) was found to be a significantly wider on the left side than the right (1.2 ± 0.7 mm) (*p* = 0.001) (Table 4).

## 4. Discussion

Blind suprascapular nerve blockade is one of the possible treatment options for both the acute and chronic pain management of suprascapular neuropathy, but the limitations of the procedure include some possible complications such as pneumothorax or injury to the neighboring vascular structures [22,23]. Ultrasound (US) visualisation of the related anatomic part and the needle itself may improve the success of the procedure and lower the complication rates [24,25].

Techniques that target the nerve more selectively may well be more advantageous. Karatas and Meray [18] have reported that nerve blocks applied close to the nerve with the assistance of electromyography (EMG) are more effective than blind injection in the suprascapular fossa. However, according to Gorthi et al. [24] ultrasound-guided suprascapular nerve block is a safe, effective, and accurate method for achieving immediate and long-term pain relief in patients with chronic, non-specific perishoulder pain, allowing for a normal range of motion, normal imaging studies, and no identified shoulder pathology.

Developments in high-frequency ultrasound transducer technology have enabled the precise investigation of several anatomical structures on small areas (nerves, vessels small band-shape fascicular structures) [15,26,27]. Ultrasonographic visualisation of the suprascapular nerve may also increase the success rate of blockage [23,24]. The use of ultrasound (US) to perform peripheral nerve blocks is a relatively new technique that is rapidly gaining popularity over the more traditional techniques.

Unlike blockades based on fluoroscopy and CT guidance, ultrasound-based nerve blockades do not require the patient and medical personnel to be exposed to harmful radiation [28]. In addition, ultrasound is known to provide significant advantages, such as its greater availability, cheapness, and repeatability. The use of ultrasound in regional anesthesia interventions may increase the success rate of the applied technique, decrease the application time, and avoid the occurrence of several probable complications [29,30]. Also, a current bibliography search indicates that ultrasonographic examination has good to excellent intra-patient, intra-examiner, and inter-examiner reliability in quantifying the peripheral nerves of the upper extremity [26]. Nevertheless, the limitation of this technique is the distance from the examined structure as in high-frequency probes the resolution decrease dramatically with increasing depth.

Suprascapular nerve block performed close to the nerve was more effective than blind injection in the suprascapular fossa. The specificity of the small area of the suprascapular region means that ultrasound plays a key role in any examination, especially when recognising the suprascapular nerve [17,19]. Visualisation of the suprascapular artery and vein is also needed to prevent unexpected bleeding during blockade procedures. A color Doppler study by Yücesoy et al. [30] identified the artery–vein suprascapular complex passing through the SSN in 86% of shoulders. In the present study, the distinction between vein and artery was made on the basis of flow spectrum analysis. Our four-step ultrasonographic protocol allowed the suprascapular artery to be found in all of the visualised suprascapular notches. In contrast, the suprascapular vein was visible only in 176 scapular notches (74.9%). It may be due to slower blood flow than in the artery, and the increased thickness of the soft tissue over the suprascapular vein, which correlated with the BMI.

According to Yücesoy et al. [30], the “skin–notch base interval” is another important parameter for nerve blockade. They predict that during the US-guided blockade, needle puncture of about 40–45 mm in length should not be exceeded so as to decrease the risk of pneumothorax and prevent periosteal pain caused by the needle at the notch base [30]. Harmon and Hearty [31] and Smoljanovic et al. [32] found the suprascapular nerve to pass through the suprascapular notch at a depth of approximately 40 mm. Our present findings indicate the thickness of the soft tissue over the suprascapular nerve, suprascapular vein, and suprascapular artery to be 36.0 ± 5.7 mm, 35.8 ± 6.0 mm, and 34.2 ± 5.7 mm, respectively. For these parameters, BMI seems to be good predictor, as significant correlations were found between BMI and the distance to artery and vein. 

The first confirmation of the value of US imaging in the identification of the suprascapular notch was reported in 1997 by Moriggl following a study of 97 volunteers; however, this interpretation was found to be difficult for partially ossified superior transverse scapular ligaments [33]. Also, Marhofer et al. [29] confirmed that the visualisation of the suprascapular nerve is limited when it is in close proximity to bony structures. Ultrasonographic investigation may recognise not only the presence, but also the shape of the suprascapular notches [17,30]. According to Polguj et al. [17], ultrasonographic examination of the SSN demonstrated high specificity for the deep shape of the SSN (97.8%), and high sensitivity in recognising its wide shape (96.9%). The suprascapular nerve was well visualised in the bottom of the suprascapular notch below the superior transverse scapular ligament.

Harmon and Hearty [31] suggested that the ideal ultrasound transducer for visualisation of the suprascapular notch region should have high-resolution capabilities between 10–15 MHz. However, ultrasound transducer resolution capabilities between 7–12 MHz have been more commonly used in previous studies [13,17,26,30,34].

Battaglia et al. [26] and Laumonerie et al. [27] reported that the trajectory of the suprascapular nerve at the level of the suprascapular notch is deep, inconsistent, and lies in the vicinity of the suprascapular artery; these factors make ultrasound-guided procedures more challenging. Our study confirms this observation. The suprascapular nerve was visualised in 63.8% of subjects. Our four-stage procedure will allow better recognition of the suprascapular artery and vein, allowing these vessels to be used as landmarks for procedures around the suprascapular notch region.

## 5. Conclusions

Our original four-step sonographic protocol enabled characterising the morphology of the SSN region despite the SSN notch type. Recognition of the SSN morphology is not affected by the BMI. Suprascapular vessels (especially the artery) are the best sonographic landmarks of the suprascapular notch region. No significant differences were found between sides regarding the thickness of the soft tissue above the suprascapular triad (neurovascular bundles). 

## Figures and Tables

**Table 1:** Distribution of the suprascapular notch types in the whole group and according to body mass index (BMI).

Type of Suprascapular Notch	All (*n* = 235) (*n*(%))	BMI (*n* (SD))	
I	26 (11.1%)	24.77 (3.49)	
II	14 (6.0%)	25.27 (3.67)	
III	151 (64.0%)	26.43 (4.02)	
IV/V	44 (18.7%)	27.38 (3.76)	
Level *p*	-	0.0536	

**Table 2:** Visualisation of the suprascapular vein and nerve.

	Suprascapular Vein (*n* = 235)	Suprascapular Nerve (*n* = 235)
Structure visible (*n*(%))	176 (74.9%)	113 (48.1%)
Structure no visible (*n*(%))	59 (25.1%)	122 (51.9%)

**Table 3:** Distribution of the sonographic measurement in the whole group, according to BMI. Differences that are significant according to Bonferroni correction are bolded.

Ultrasonographic Measurements	All (*n* = 235)	BMI	
		R2	Level *p*
Superior transverse diameter (mm)	14.3 (4.4)	0.0194	0.4881
Maximal depth (mm)	6.5 (2.2)	0.0040	0.7537
Diameter of suprascapular artery (mm)	1.8 (0.7)	0.0009	0.8803
Thickness of the soft tissue over the suprascapular artery (mm)	34.2 (5.7)	0.3196	0.0021
Diameter of suprascapular vein (mm)	1.4 (0,8)	0.0779	0.1585
Thickness of the soft tissue over the suprascapular vein (mm)	35.8 (6.0)	0.2281	0.0118
Diameter of suprascapular nerve (mm)	3.3 (1.0)	0.0034	0.7711
Thickness of the soft tissue over the suprascapular nerve (mm)	36.0 (5.7)	0.1052	0.0988

**Table 4:** Comparison of sonographic measurements between body sides. Differences that are significant according to Bonferroni’s correction are bolded.

Ultrasonographic Measurements	Right Side (*n* = 116)	Left Side (*n* = 119)	Level *p*
Superior transverse diameter (mm)	14.8 ± 4.8	13.8 ± 4.0	0.0332
Maximal depth (mm)	6.3 ± 2.1	6.6 ± 2.3	0.3586
Diameter of suprascapular nerve (mm)	3.5 ± 1.1	1.3 ± 0.4	0.0010
Thickness of the soft tissue over the suprascapular nerve (mm)	38.0 ± 5.2	37.7 ± 6.1	1.0000
Diameter of suprascapular artery (mm)	1.8 ± 0.7	1.8 ± 0.7	0.5904
Thickness of the soft tissue over the suprascapular artery (mm)	34.6 ± 5.7	33.6 ± 5.7	0.0202
Diameter of suprascapular vein (mm)	1.2 ± 0.7	1.5 ± 0.9	0.0010
Thickness of the soft tissue over the suprascapular vein (mm)	36.2 ± 5.9	35.2 ± 6.3	0.0147

## Data Availability

The data used to support the findings of this study are available from the corresponding author upon request.
